# Bevacizumab treatment induces metabolic adaptation toward anaerobic metabolism in glioblastomas

**DOI:** 10.1007/s00401-014-1352-5

**Published:** 2014-10-17

**Authors:** Fred Fack, Heidi Espedal, Olivier Keunen, Anna Golebiewska, Nina Obad, Patrick N. Harter, Michel Mittelbronn, Oliver Bähr, Astrid Weyerbrock, Linda Stuhr, Hrvoje Miletic, Per Ø. Sakariassen, Daniel Stieber, Cecilie B. Rygh, Morten Lund-Johansen, Liang Zheng, Eyal Gottlieb, Simone P. Niclou, Rolf Bjerkvig

**Affiliations:** 1 NorLux Neuro-Oncology Laboratory, Department of Oncology, Centre de Recherche Public de la Santé, Strassen, Luxembourg; 2 NorLux Neuro-Oncology, Department of Biomedicine, University of Bergen, Jonas Lies vei 91, 5019 Bergen, Norway; 3 Edinger Institute, Institute of Neurology, Goethe University, Hospital Frankfurt, Frankfurt am Main, Germany; 4 Dr. Senckenberg Institute of Neurooncology, Goethe University, Hospital Frankfurt, Frankfurt am Main, Germany; 5 Department of Neurosurgery, University Hospital Freiburg, Freiburg, Germany; 6 Matrix Biology Group, Department of Biomedicine, University of Bergen, Bergen, Norway; 7 Department of Pathology, Haukeland University Hospital, The Gade Institute, Bergen, Norway; 8 Department of Biomedicine, Molecular Imaging Center, University of Bergen, Bergen, Norway; 9 Department of Neurosurgery, Haukeland University Hospital, Bergen, Norway; 10 Cancer Research UK, Beatson Institute, Glasgow, Scotland, UK; 11 KG Jebsen Brain Tumour Research Center, University of Bergen, Bergen, Norway

**Keywords:** Glioblastoma, Bevacizumab, Metabolism, Adaptation

## Abstract

Anti-angiogenic therapy in glioblastoma (GBM) has unfortunately not led to the anticipated improvement in patient prognosis. We here describe how human GBM adapts to bevacizumab treatment at the metabolic level. By performing ^13^C_6_-glucose metabolic flux analysis, we show for the first time that the tumors undergo metabolic re-programming toward anaerobic metabolism, thereby uncoupling glycolysis from oxidative phosphorylation. Following treatment, an increased influx of ^13^C_6_-glucose was observed into the tumors, concomitant to increased lactate levels and a reduction of metabolites associated with the tricarboxylic acid cycle. This was confirmed by increased expression of glycolytic enzymes including pyruvate dehydrogenase kinase in the treated tumors. Interestingly, l-glutamine levels were also reduced. These results were further confirmed by the assessment of in vivo metabolic data obtained by magnetic resonance spectroscopy and positron emission tomography. Moreover, bevacizumab led to a depletion in glutathione levels indicating that the treatment caused oxidative stress in the tumors. Confirming the metabolic flux results, immunohistochemical analysis showed an up-regulation of lactate dehydrogenase in the bevacizumab-treated tumor core as well as in single tumor cells infiltrating the brain, which may explain the increased invasion observed after bevacizumab treatment. These observations were further validated in a panel of eight human GBM patients in which paired biopsy samples were obtained before and after bevacizumab treatment. Importantly, we show that the GBM adaptation to bevacizumab therapy is not mediated by clonal selection mechanisms, but represents an adaptive response to therapy.

**Electronic supplementary material:**

The online version of this article (doi:10.1007/s00401-014-1352-5) contains supplementary material, which is available to authorized users.

## Introduction

In recent years, various -omics and deep sequencing technologies have provided important new insight into molecular sub-classifications of GBMs that have led to better prognostic information. However, less is known about the mechanisms of adaptability of tumor cells to changes in the microenvironment and how these changes affect the tumor cell populations. Such changes may either arise due to alterations in the microenvironment during tumor progression, or as a response to therapy. During the last few years, the vascular endothelial growth factor (VEGF) inhibitor bevacizumab has been extensively applied for the treatment of recurrent GBMs. Yet, recent results from two double-blind, placebo-controlled phase III trials, AVAglio [6] and RTOG 0825 [15], show that bevacizumab treatment does not increase overall survival, but leads to a minor increase in progression-free survival. GBMs are characterized by their diffuse infiltrative growth as well as extensive angiogenesis where VEGF has been shown to play a major role [45]. Thus, from a theoretical viewpoint, blocking the formation of new blood vessels should be beneficial for GBM patients and, indeed, dramatic effects are seen, exemplified by a strong reduction in contrast enhancement (CE) as well as vessel diffusion and perfusion, assessed by magnetic resonance imaging (MRI) following treatment [2, 8, 9, 12, 54, 57]. However, the exact mechanism by which GBMs evade bevacizumab treatment still remains enigmatic. Nevertheless, during the last decade intensive research has led to new insight into putative escape mechanisms. This includes: (i) escape via different modes of vascularization mediated by sprouting angiogenesis, vasculogenesis, vessel co-option and vascular mimicry (reviewed in [32, 51]); (ii) recruitment of pro-angiogenic myelogenic cells from the circulation [44]; (iii) secretion of numerous alternative pro-angiogenic factors such as bFGF, PIGF, Ephrins, PDGF-C, SDF-1α (reviewed in [33] and others [21, 31, 35]. Also, increased glioma cell invasion has been seen following suppressed VEGF signaling [8, 27, 47, 48]. For instance, a relative hostile hypoxic microenvironment induced by anti-angiogenic therapy may lead to an up-regulation of pro-invasive programs characterized by c-MET/HGF-induced mesenchymal transformation [24, 43], β1-integrin up-regulation [23] and increased MMP expression [13]. To add to this complexity, we have previously shown in a human GBM-derived xenograft model (that recapitulates the genotype and phenotype of the corresponding patient tumors) that vascular remodeling induced by anti-VEGF treatment leads to a more hypoxic tumor microenvironment which appears to favor metabolic changes in the tumors toward glycolysis, and where the net result is increased tumor cell invasion into the normal brain [27]. To study how bevacizumab affects the GBM metabolism, we performed ^13^C_6_-glucose injections into tumor-bearing animals, followed by LC–MS based metabolomic analysis to determine in detail metabolic flux and steady-state metabolites in tumor extracts after treatment. To assess tumor growth, we also used complementary in vivo imaging methods including MRI and PET. Moreover, to determine if the observed changes in metabolism were caused by an adaptive response or clonal selection events within the tumors, we performed a flow cytometric ploidy analysis and multiparameter phenotyping on treated and non-treated tumors.

## Materials and methods

### Tumor material

Organotypic biopsy spheroids from human GBMs were prepared as previously described [4, 27] Briefly, tumor samples were minced into ~0.2 mm fragments and placed into tissue culture flasks base coated with 0.75 % agar (Difco, Detroit, MI, USA). The spheroids were maintained on agar pre-coated flasks in spheroid medium (DMEM medium, 10 % FBS, 2 mM l-glutamine, 0.4 mM NEAA and 100 U/ml Pen-Strep) for 7–10 days, in a standard tissue culture incubator with 5 % CO_2_ and 100 % relative humidity at 37 °C. The spheroids used in the present study were derived from two GBM patients (P3 and P13) and expanded through serial transplantations in Rowett nude rats (RNU). This generated a standardized pool of spheroids (300–400 µm in diameter) giving rise, after orthotopic xenografting, to highly invasive and angiogenic tumors [58] that show a similar DNA copy number as seen in human GBMs (Supplementary Fig. 1). The collection of human biopsy tissue was approved by the regional ethical committee at Haukeland University Hospital, Bergen, Norway (REK 013.09).

### Intracranial implantation

The P3 and P13 spheroids were stereotactically implanted into the brains of nude rats (*n* = 28 for P3 and *n* = 11 for P13) as well as in eGFP NOD/SCID mice (*n* = 5 for P3) as described previously [16, 40, 58] Briefly, GBM spheroids (10 per rat, 6 per mouse) were implanted into the right cerebral cortex using a Hamilton syringe fitted with a needle (Hamilton, Bonaduz, Switzerland). The handling of the animals and the surgical procedures were performed in accordance with the Norwegian Animal Act and the European Directive on animal experimentation (2010/63/EU) and the local ethical committee approved the protocol.

### Bevacizumab treatment and sacrifice

**Fig. 1:**
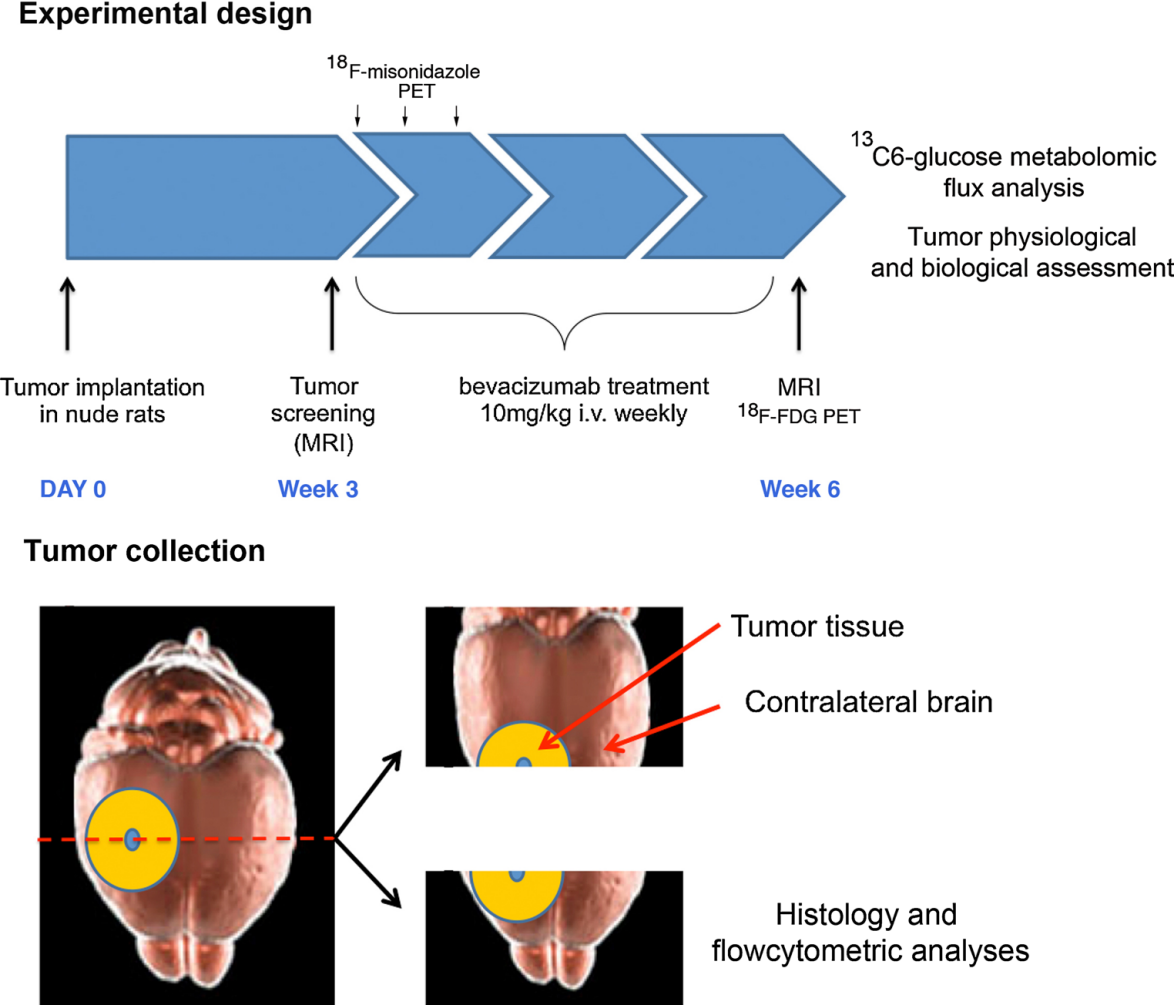
Schematic representation of the experimental design. Three weeks after implantation, tumor growth was assessed by MRI. The rats were then treated weekly with bevacizumab (10 mg/kg) for 3 weeks. During the first week of treatment, ^8^F-FMISO and ^18^F-FDG PET were performed at day 0, 3 and 7. At week 6, the animals underwent MRI. Before killing, the animals were infused i.v with ^13^C_6_-glucose, whereupon the brains were harvested at 15 min at 120 min after infusion for further analysis. For the flux analysis, tumor tissue was collected both from the tumor core as well as from the contralateral brain (*lower panel*)

Tumor take in rats was verified by MRI 3 weeks post-implantation and animals were stratified into control (*n* = 13 for P3 and *n* = 6 for P13) and treatment groups (*n* = 15 for P3 and *n* = 5 for P13). Animals from the treatment group received one weekly i.v injection of bevacizumab (10 mg/kg) into the tail vein for 3 weeks. The control animals received saline following the same schedule. When symptoms developed, the rats were examined by MRI and subsequently killed; the brains were dissected out and cut into two pieces along the coronal plane at the tumor core. One piece was collected and fixed for histological and immunohistological analyses (overnight fixation in 4 % PFA) and the other was snap frozen in liquid N_2_ for ^13^C_6_-glucose metabolic flux analysis. For the flux analysis, tumor tissue was collected from the tumor core as well as from the contralateral brain. Eight non-tumor bearing animals were divided into a treatment and a control group as above (*n* = 4, both groups) and treated for 3 weeks accordingly before killing and tissue processing (see Fig. 1 for details). The GFP-expressing NOD/Scid mice received weekly intraperitoneal injections of bevacizumab (20 mg/kg in saline), starting 3 weeks after spheroid implantation. Control animals received injections of saline. The mice were killed on appearance of neurological symptoms.

### Western blot analyses

Snap-frozen tumor tissue extracts (tumor cells confirmed by HE-staining) were put in Kinexus Lysis Buffer and homogenized using a pellet pestle motor. Protein concentrations were determined using the BCA Protein assay kit (Pierce, BioRad, Oslo, Norway). Proteins (20–25 μg) were separated by SDS/PAGE using NuPage precast gels (Invitrogen) and transferred to nitrocellulose membranes. Membranes were blocked for 1 h at RT in blocking buffer (5 % milk powder or 5 % BSA in TBS with 0.1 % Tween-20) and incubated overnight at 4 °C, with anti-LDHA diluted 1:500 (Cell Signaling Technology, #3582, Leiden, the Netherlands), anti-PFKP 1:700 (Cell Signaling, #8164), anti-TKT 1:700 (Cell Signaling, #8616), anti-HKII 1:500 (Abcam, #ab37593, Cambridge, UK), anti-ALDOC 1:500 (Sigma-Aldrich, #HPA00328, Oslo, Norway), anti-PDK1 1:1500 (Sigma-Aldrich, #HPA027376) and anti-β-actin 1:1000 (Abcam #ab8227) in blocking buffer. The primary antibodies were detected using an HRP-conjugated goat anti-rabbit secondary antibody (Immunotech, #IM0831, Brea, CA, USA) diluted 1:20000. The blots were developed using Pierce Supersignal West Femto Chemiluminescent Substrate (Pierce Biotechnology, Rockford, IL, USA) and detected with a Fuji LAS 3000 Imager (Fuji Life Science, Dusseldorf, Germany). The blots were quantified using Fuji Multi Gauge software (Fuji Life Science) and analyzed (*n* = 2) with unpaired independent-samples *t* test (Excel software). Statistical significance was set at two levels, *p < 0.1 and **p < 0.05.

### Immunohistochemistry

Paraffin-embedded formalin-fixed tissue sections were deparaffinized and heated at 99 °C for 20 min in 10 mM citrate buffer at pH 6.0 or incubated with proteinase K diluted in 0.05 M Tris–Cl, pH 7.5 at 37 °C for 10 min. The sections were incubated with the following primary antibodies: anti-human nestin (MAB5326; Millipore; 1:1000 Billerica, MA, USA), anti-von Willebrand factor (A0082; DAKO; 1:1000, Oslo, Norway), anti-LDHA (3582; Cell Signaling, 1:200) and pimonidazole (Hypoxyprobe 9.7.11, HPI Inc, 1:200, Burlington, MA, USA). Primary antibodies were incubated for 90 min at RT. Detection was performed using a biotinylated secondary antibody (Vector Laboratories, Trondheim, Norway) amplified with Vectastain ABC Reagent (Vector). Sections were developed using 3′3-diaminobenzidine (DAB, DAKO), following the manufacturer’s instructions. The quantification of vessel area fractions was analyzed in five microscopic high power fields for each section. Quantification of invasion was performed by delineating the tumor core and counting nestin-positive cells outside the tumor core. Analysis was performed on five microscopic high-power fields for each section. The immunohistochemical stainings were analyzed and pictures were taken with a Nikon light microscope (Nikon Eclipse E600, Melville, NY, USA) using Nikon imaging software (Nikon NIS Elements v 4.11).

### Determination of NADP^+^/NADPH ratio

For the relative quantification of the oxidized and reduced adenine dinucleotide phosphates (NADP^+^ and NADPH) in tissue extracts, we used a bioluminescent assay NADP^+^/NADPH-Glo™ Assay (Promega, G9081). Luminescence signals were measured in a 96-well format in a LUMIstar plate reader. Total oxidized and reduced nicotinamide amounts expressed in relative luminescence units served to determine NADP^+^/NADPH ratios in tissue extracts. Calibration of the assay was done with an NADP+ standard curve. For comparative analysis we used a standardized extraction procedure as described by the manufacturer (25 μl extraction buffer per milligram of tissue).

### MRI

Anatomical MRI sequences were acquired on a 7T Pharmascan (Bruker Biospin, Ettlingen, Germany), equipped with a quadratic rat head transmit/receive coil. Animals were anesthetized with 1–2 % isoflurane mixed with 50 % air and 50 % O_2_ and placed in a prone position in a cradle with a heating pad set at 37 °C. Breathing was monitored throughout the scanning sessions. Anatomical sequences used included T2-weighted (fast spin echo, TE/TR: 36 ms/3,500 ms, in-plane resolution: 0.137 × 0.137 mm per pixel, slice thickness: 1 mm) as well as T1-weighted (fast spin echo, TE/TR: 7.5 ms/1,300 ms, in-plane resolution: 0.137 × 0.137 mm per pixel, slice thickness: 1 mm) before and after injection of a contrast agent (1 ml of undiluted gadodiamide, Omniscan, GE Healthcare, Stockholm, Sweden at 0.5 mmol/ml injected s.c). Magnetic resonance spectroscopy (MRS) data were acquired by ^1^H PRESS (TE/TR: 5.9 ms/2,500 ms in a single 3 × 3 × 3 mm^3^ voxel centered in the tumor, 256 averages) and analyzed with LCModel as previously described [27].

### Positron emission tomography (PET) imaging

^18^F was produced by a local cyclotron (GE PETrace 840, Uppsala, Sweden) and used to synthesize ^18^F-fluorodeoxyglucose (^18^F-FDG) and ^18^F-fluoromisonidazole (^18^F-FMISO) using kits from ABX GmBH, Radeberg, Germany. PET/CT sequences were acquired on a dedicated CT 80 W small animal Nanoscan PC imager from Mediso Medical Imaging Systems, Budapest, Hungary. CT X-ray images were acquired for animal positioning, anatomical reference and attenuation correction during PET data reconstruction, using the following parameters: helical trajectory, tube voltage: 50 kVp, exposure time: 300 ms, 480 projections, binning 1:4 and matrix size 250 × 250 × 250 μm. Accumulation of ^18^F-FDG was measured for 40 min following the injection of 13.8 ± 1.3 MBq of activity. Injections of ^18^F-FMISO were performed the following day. 29.3 ± 3.8 MBq of activity was injected, after which the animal was left awake for 120 min before the start of a 30 min acquisition scans. This delay of 120 min had, in preliminary dynamic studies, been found to provide good tracer accumulation and tumor-to-brain tissue contrast. Activity doses were diluted in a saline solution and a volume of 1 ml was injected into the tail vein. Animals were anesthetized using 2.5 % sevoflurane mixed in air for the duration of the scans and monitored for breathing and body temperature. Mediso Nucline software was used for PET data reconstruction from listmode using the following parameters: reconstruction algorithm 3D OSEM (6 iterations), energy window 400–600 keV, coincidence mode 1–5, corrections for random events, detector normalization, decay and dead time and voxel size 0.6 mm. Mediso Inter View Fusion software (version 2.02.055.2010) was used for data visualization, co-registration of PET and CT data and quantification of standard uptake values as in [59].

### Metabolic flux analysis

#### U-^13^C_6_-glucose administration and definition of sampling time

U-^13^C_6_-glucose (Cambridge Isotope Laboratories, Tewksbury, MA, USA) was injected at a dose of 0.5 ml/100 g body weight using a 200 mg/ml glucose solution in 1× PBS for a single bolus injection in the tail vein. Blood samples of three normal rats were collected after 15, 30, 60 and 120 min and the serum concentrations of ^13^C_6_-glucose were determined by LC–MS analysis (Supplementary Fig. 2). Based on this information, we decided to collect tissue samples after 15 and 120 min. In non-implanted control rats, brain tissue from the 15 min time point was collected.

#### Tissue and blood collection

The xenografts were immediately dissected out and cut in a frontal and posterior part at the site of implantation. One part of the brain was cryopreserved (transferred in isopentane in liquid N_2_ immersion) or formalin fixed for histology. From the second part, tumor and contralateral brain tissue fragments were dissected (average weight 93 mg) and transferred into cryotubes and frozen in liquid N_2_. For the animals without tumor, implantation tissues from both hemispheres were collected in the same manner. Blood samples were also taken intracardially at killing from all animals and maintained on dry ice in vacutainers before transfer to −80 °C.

#### Metabolite extraction

The collected tissue fragments were subdivided on ice in smaller fragments (10–20 mg) and transferred in 2 ml Eppendorf tubes for metabolite or nucleic acid extractions. For metabolite extraction, a 5 mm metal bead (Qiagen, No69989) and extraction buffer (methanol/acetonitrile/water-50/30/20) containing 100 ng/ml HEPES for internal standard purpose (Sigma, H4034) was added to the tissue at a volume/weight ratio of 25 μl/mg. The tissue was disrupted in a bead mill (Qiagen, Tissuelyzer, Hombrechtikan, Switzerland) with two cycles (2 min/20 MHz) before vortexing on an Eppendorf Thermomixer at 2 °C/20 min/1,400 rpm. The extract was clarified by centrifugation (15 min/12k rpm/4 °C in an Eppendorf centrifuge) and transferred into fresh tubes for storage at −80 °C for LC–MS.

### LC–MS analysis

The column used was the ZIC-pHILIC (150 × 2.1 mm id 5 μm; SeQuant) with the guard column (20 × 2.1 mm id 5 μm; Hichrom). Mobile phase A: 20 mM ammonia carbonate plus 0.1 % ammonia hydroxide in water. Mobile phase B: acetonitrile. The flow rate was kept at 100 μl/min and gradient as follows: 0 min 80 % of A, 30 min 20 % of B, 31 min 80 % of B and 45 min 80 % of B. The Exactive Orbitrap mass spectrometer (Thermo Scientific, Waltham, MA, USA) was operated in a polarity switching mode. All solvents were of HPLC grade purity.

### Gene expression analysis

Total RNA was extracted using the RNeasy Plus Mini Kit (QIAGEN^®^, Germantown, MD). 1 μg of total RNA was reverse transcribed using iScript cDNA synthesis Kit (Biorad) according to the manufacturer’s instructions and real-time quantitative PCR (Q-PCR) was carried out using TaqMan^®^ Fast Advanced Master Mix and the Viia™ 7 Real Time System (Applied Biosystems). See [26] for the oligonucleotides used. The amplification temperature was kept at 60 °C. Cycle threshold (*C*_t_) values were determined in the exponential phase of the amplification curve and the ΔΔCT method was used for fold change calculations (QBase software). RPLI3A was used as a housekeeping gene. Four (P13) to five (P3) animals per group were used and all samples were run in triplicate. Data were analyzed with unpaired independent-samples *t* test (Excel software). Statistical significance was set at two levels, *p < 0.1 and **p < 0.05.

### Patient data

We investigated paraffin sections from eight GBM patients at two time points. The first set of samples was obtained after primary surgery and the second after bevacizumab treatment (5 autopsy and 3 reoperations). LDH expression was examined by means of immunohistochemistry (for detailed patient characteristics and time of biopsy collection after bevacizumab treatment see (Supplementary Table I). The study was approved by the local ethical committee of the Goethe University Frankfurt, Germany (GS 4/09; SNO_10-13).

### Immunohistochemistry of patient biopsies

Immunohistochemistry on the tumor specimens was performed using an automated staining system Discovery XT (Roche/Ventana, Tucson, USA) as previously described [3]. Rabbit anti-human LDHA antibody (Cell Signaling #3582) was used as primary antibody.

### Statistical analysis

The proportion of LDH-positive vs -negative cells in the GBM samples described above was determined by counting all cells using the Stereo Investigator as well as the Fractionator (MicroBrightField Inc, Williston, USA), allowing for an unbiased evaluation of tumor areas. Counts from matched pairs of primary and recurrent post-bevacizumab tumors were analyzed using a paired *t* test. The same test was used to compare data from the metabolic flux analyses. Statistical significance of changes in MRS-identified lactate concentration was assessed by analysis of variance routines in Matlab (Mathworks, Natick, MA, USA) using the tumor volume and treatment as independent factors.

### Ploidy analysis combined with cell membrane phenotyping

Flow cytometry experiments were performed as described before [16, 17]. Briefly, xenografts derived in eGFP-expressing mice were minced with scalpels and dissociated with MACS Neural Tissue Dissociation Kit (P) (Miltenyi, 130-092-628, Lund, Sweden) following the manufacturer’s instructions. Single cell suspensions were incubated with Hoechst 33342 (5 µg/ml, Bisbenzimide, Ho342; Sigma) at 37 °C in pre-warmed DMEM, containing 2 % FBS, 10 mM HEPES pH 7.4 and DNAse I (10 µg/ml; Sigma) at 1 × 10^6^ cells/ml for 120 min. After washing, cells were resuspended in ice-cold HBSS 2 % FBS and 10 mM HEPES pH 7.4 buffer (100 µl/test). Prior to flow cytometry, cells were incubated with LIVE/DEAD^®^ Fixable Dead Cell Stains (Life Technologies) and appropriate preconjugated antibodies for 30 min at 4 °C in the dark (antibodies are listed in Supplementary Table II). Data acquisition was performed on a FACS Aria™ SORP cytometer (BD Biosciences, San Jose, CA, USA) and the Hoechst signal was excited with the UV laser. Data acquisition and analysis were done with DIVA software (BD Biosciences). Histograms were prepared with the FlowJo software.

## Results

### Bevacizumab induces reduction of contrast enhancement and normalization of vascular morphology

**Fig. 2:**
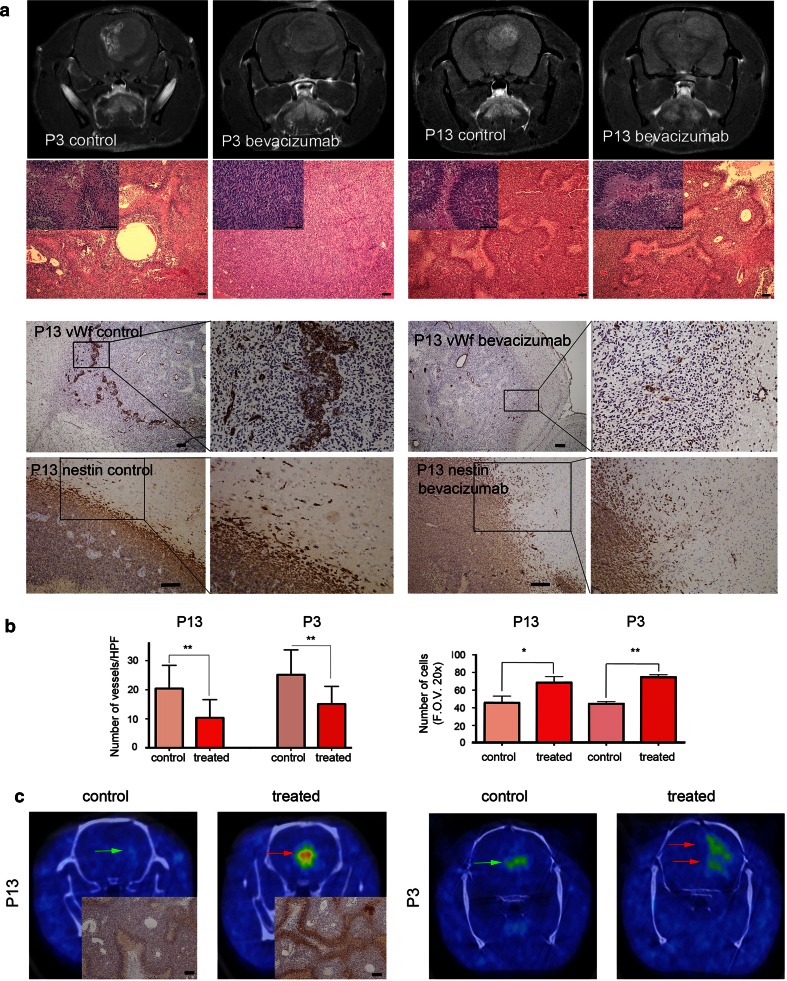
Bevacizumab induces reduction of contrast enhancement and normalization of vascular morphology. **a** *Upper panels*: T1 contrast-enhanced MRI images of control and bevacizumab-treated P3 and P13 tumors at week 6 after implantation. A reduction in contrast enhancement is seen after bevacizumab treatment. Corresponding H&E-stained sections of P3 tumors showing a reduction in pseudopalisading necrosis (*insets*), whereas this was not observed in the P13 tumors. (*bars* 100 µm). *Lower panels*: vWf factor staining of blood vessels in control P13 tumors showing a strong proliferation of endothelial cells at the tumor margin as indicated by numerous vascular nests. These nests were absent in the bevacizumab-treated tumors (*bars* 100 µm). P13 tumors stained with a nestin human monoclonal antibody show an increased invasion in bevacizumab-treated tumors compared to controls (*bars* 100 µm). **b** A significant reduction in blood vessels was observed for both P13 and P3 tumors following bevacizumab treatment, whereas in both tumors a significant increase in tumor cell invasion was seen. **c** ^18^F-FMISO PET images after the first week of bevacizumab treatment revealed a stronger hypoxia signal in both P3 and P13 tumors in the treatment groups (*red arrows*) compared to untreated control tumors (*green arrows*). For the P13 tumors, the ^18^F-FMISO PET images were confirmed by immunostaining for the hypoxyprobe pimonidazole (*insets*; *bar* 100 µm)

We have previously established a human GBM xenograft system that at the level of DNA copy number variation closely reflects the corresponding human tumors in situ [49, 58]. Of the two GBMs used here, one (P3) was of mesenchymal subtype and the other (P13) of neural subtype. P3 shows trisomy of Chr7, Chr19, 20q, homozygous deletion of 1q42-q43, Chr9, Chr10, 20p and loss of PIK3R1 and CDKN2A/B. P13 harbors trisomy of Chr7, Chr19, Chr20, homozygous deletion of 6q16.2–16.3, Chr10, 17q12 and loss of CDKN2A/B (Supplementary Fig. 1). The untreated P3 and P13 xenografts showed typical hallmarks of GBMs as indicated by pseudopalisading necrotic areas and microvascular proliferations. After bevacizumab treatment, MRI confirmed observations obtained from numerous clinical as well as experimental studies showing a reduction in contrast enhancement (Fig. 2a, upper panel). Interestingly, histological analysis of the bevacizumab-treated tumors revealed a reduction of necrotic areas in the P3 tumors, whereas in the P13 tumors, extensive necrosis was observed (Fig. 2a, upper panel). The vascular architecture in the tumors was assessed by von Willebrand factor (vWF) antibody staining. In both tumors, a vascular normalization at the structural level was observed (Fig. 2a, lower panel). We have previously described the blood vessel architecture, following bevacizumab treatment for P3 tumors [27]. For the P13 xenografts, also a strong reduction in areas of endothelial proliferation was observed (Fig. 2b). As previously shown by us [27] and other groups [8, 47, 48], a significant increase in the number of tumor cells invading the normal brain was observed after bevacizumab treatment for both xenografts (Fig. 2a, lower panel and Fig. 2b). In summary, these results verify to a large extent results obtained from preclinical as well as clinical bevacizumab treatment studies.

### Hypoxia is induced in the tumors after bevacizumab treatment

We have previously indicated that HIF1α is up-regulated in P3 tumors after bevacizumab treatment [27]. ^18^F-FMISO PET imaging on both xenograft models (*n* = 2 and *n* = 3 per group for P3 and P13, respectively), at 1, 3 and 7 days, following treatment, revealed a strong signal increase in the treatment groups compared to controls during the 7 day treatment period, suggesting that bevacizumab causes a gradual increase of hypoxia in the tumors (Fig. 2c). Thus, it appears that bevacizumab leads to reduced oxygenation in the tumor at early (shown here) and late time points [27]. In this context, a vascular normalization window at a functional level, which implies improved oxygenation and reduced hypoxia, was not observed by ^18^F-FMISO PET during the first 7 days of treatment (Fig. 2c).

## ^13^C_6_-glucose metabolic flux analysis shows increased glycolysis after bevacizumab treatment

**Fig. 3:**
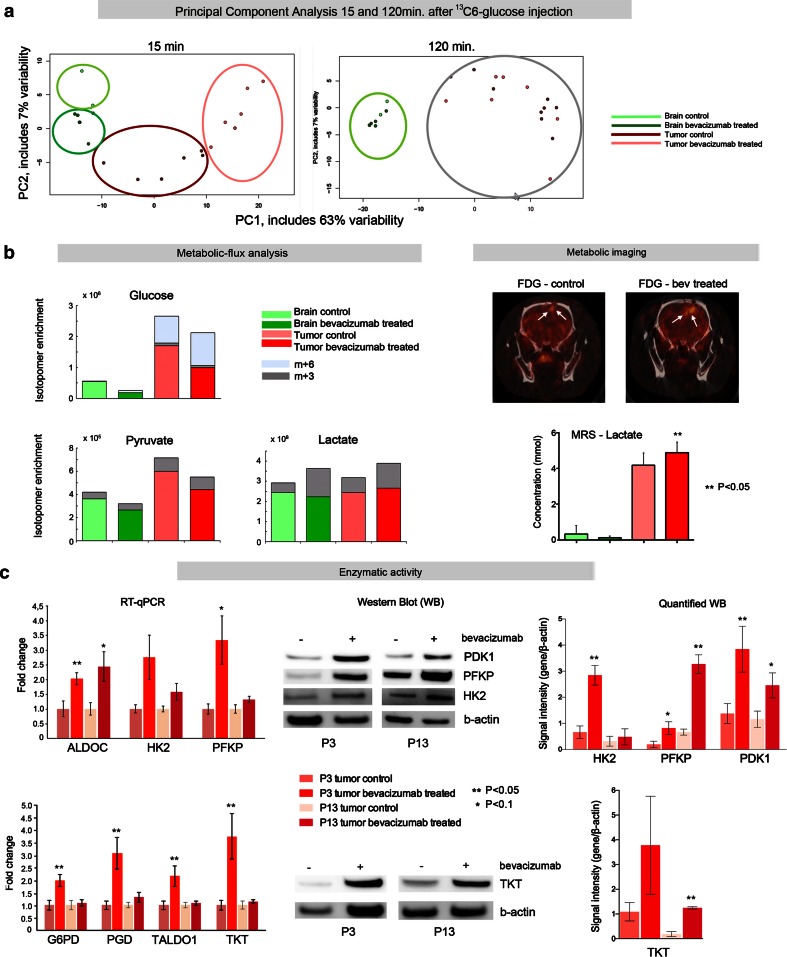
Increased glycolytic activity following bevacizumab treatment. **a** Principal components analysis (PCA) revealed clustering of metabolites extracted at 15 min after ^13^C_6_-glucose injection in the different animal groups: control and bevacizumab-treated tumors and contralateral brain (*left panel*). Distinctions between treated and control groups were, however, not evident at 120 min after injection (*right panel*). **b** *Left panel*: metabolic ^13^C_6_ glucose carbon flux analysis of labeled (*gray*) and unlabeled (colored) metabolites. The ^13^C isotopologues reveal an increased influx of labeled glucose (m + 6) leading to an increase of labeled pyruvate (m + 3) and lactate (m + 3) in the bevacizumab treatment tumors compared to the controls (*dark gray bars*: m + 3 isotopologues, *light gray bars*: m + 6 isotopologs). Increased labeled lactate was also seen in the contralateral brain upon treatment. Note that levels of unlabeled ^12^C glucose and pyruvate were reduced in treated tumors, while unlabeled lactate levels were increased, suggesting a depletion of the glucose pool in favor of lactate production (*pink bars*: untreated tumor, *red bars*: treated tumors). *Right panel*: ^18^F-FDG micro-PET imaging showing an increased uptake of this radioactive glucose analog after treatment (*arrowheads* depict the ^18^F-FDG signal). In vivo MRS shows a 17 % increase of lactate in the bevacizumab-treated animals confirming the results of the metabolic flux analysis. **c** Expression analysis of key metabolic enzymes. *Left panel*: RT-qPCR analysis shows an up-regulation of glycolytic enzymes (ALDOC, HK2 and PFKP) as well key enzymes of the pentose phosphate pathway (PPP) (G6PD, PGD, TALDO1 and TKT), in both P3 and P13 xenografts. Yet there is a stronger trend in P3 tumors compared to P13. Western blots (*central panel*) substantiated these observations indicating an up-regulation of PDK1, PFKP, HK2 and TKT protein after treatment. *Right panel*: quantification of the blots after normalization to β-actin

We have previously shown that bevacizumab induces higher levels of lactate in P3 tumors, suggesting an increase in glycolytic activity [27]. We therefore performed a detailed metabolic flux analysis of the P3 xenografts. First, we infused ^13^C_6_-glucose i.v into normal rats and collected blood samples at 15, 20, 60 and 120 min after infusion. The blood samples were then subjected to LC–MS analysis where both ^13^C-labeled and unlabeled glucose were quantified. These initial studies revealed a rather quick utilization of glucose in the rats (Supplementary Fig. 2). Based on these results, we harvested the brain 15 and 120 min after ^13^C_6_-glucose injection for metabolomic analysis. Tissues were collected from the main tumor mass as well as from the contralateral normal brain (Fig. 1). For metabolic analysis, metabolites were extracted from tumors and contralateral brain samples and quantified by LC–MS ion counts. The data of the metabolite M + 0 mass isotopologue and of M + 6 glucose isotopologue were then normalized and analyzed by principal components analysis (PCA). Clustering of samples between brain and tumor, and between treated and control animals were evident 15 min after labeled glucose injection (Fig. 3a, left panel). Distinctions between treated and control groups in brain or tumor samples were, however, less evident 120 min after injection (Fig. 3a, right panel).

A detailed analysis of metabolites 15 min after the injection of labeled ^13^C glucose revealed increased levels of labeled glucose (m + 6) in the tumors of bevacizumab-treated animals (Fig. 3b; left panel), suggesting an increased glucose flux after treatment. In agreement with this (increased glucose consumption), unlabeled ^12^C glucose and pyruvate were lower in the tumors of bevacizumab-treated animals compared to control tumors. At the same time total lactate levels (both labeled and unlabeled) were significantly increased after treatment, indicating an uncoupling of glycolysis from oxidative phosphorylation in favor of lactate production (Fig. 3b; left panel). An increased production of labeled lactate was also observed in the healthy brain which may suggest an effect of bevacizumab on normal brain cells or a contamination of the tissue with infiltrating tumor cells.

The observations of increased glucose consumption were further substantiated by ^18^F-FDG micro-PET imaging showing an increased uptake of this radioactive glucose analog after treatment (Fig. 3b; right upper panel). As expected, concentrations of lactate measured in vivo by MRS showed a 17 % increase in the bevacizumab-treated animals (*n* = 15) compared to the control group (*n* = 17), confirming the results of the flux analysis (Fig. 3b; right lower panel).

We also assessed, after bevacizumab treatment, changes in expression of key enzymes associated with glycolysis and with the pentose phosphate pathway (PPP) in both the P3 and P13 xenografts. As shown in Fig. 3c, the glycolytic enzymes aldolase C (ALDOC), hexokinase 2 (HK2) and phosphofructokinase 1 (PFKP), as well as the PPP enzymes glucose-6-phosphate dehydrogenase (G6PD), phosphogluconate dehydrogenase (PGD), transaldolase 1 (TALDO1) and transketolase (TKT) were significantly up-regulated at the transcriptome level, yet with a stronger trend in the P3 tumors compared to P13 (Fig. 3c, left panels). Of note, the enzymes G6PD and PGD are both NADPH-producing enzymes in the oxidative arm of the PPP which may contribute to NADPH production in the tumors. Increased enzyme levels were further confirmed at the protein level for PDK1, PFKP, HK2 and TKT (Fig. 3c, middle and right panels).

In summary, the presented results provide definitive evidence that bevacizumab treatment leads to an up-regulation of glycolysis, concomitantly with an activation of the PPP pathway which may represent a major carbon source for energy and biomass production in the treated tumors.

### Bevacizumab treatment causes a reduction of metabolites associated with the tricarboxylic acid cycle (TCA cycle)

**Fig. 4:**
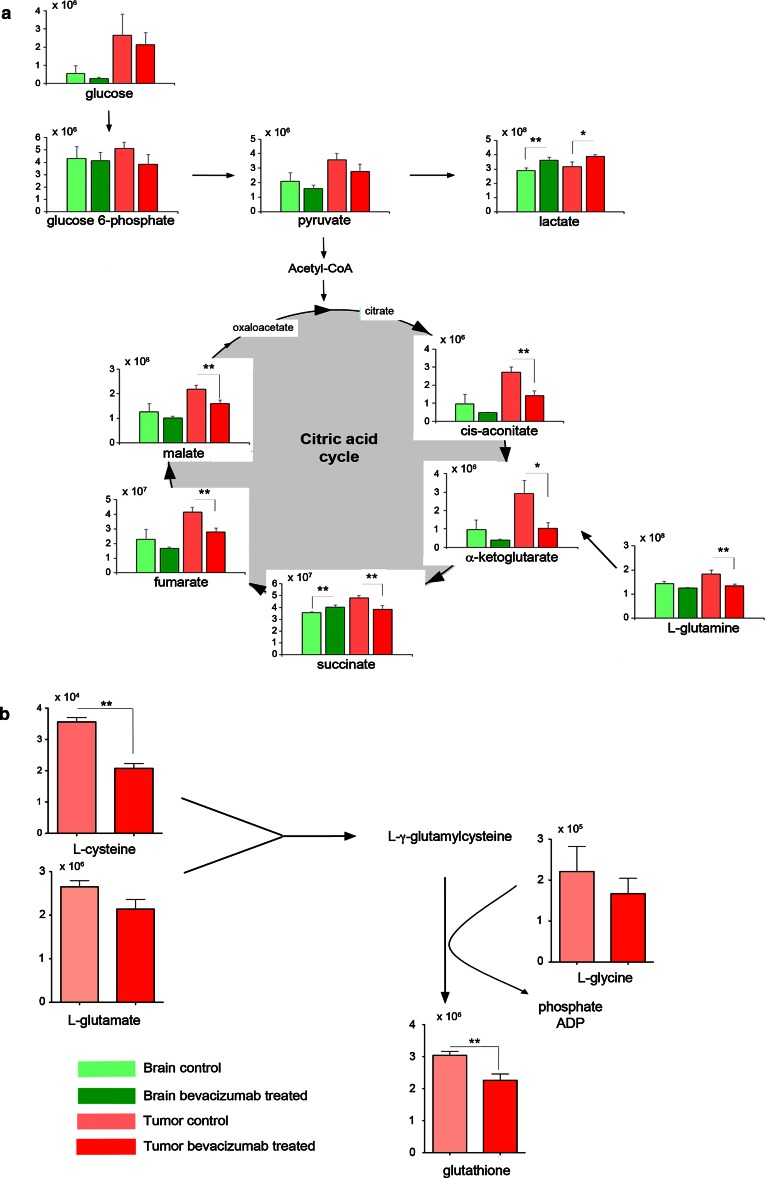
Bevacizumab treatment causes a reduction of metabolites associated with the TCA cycle. **a** Total metabolite levels (unlabeled and labeled) were quantified by LC–MS analysis. In addition to decreased glucose, glucose-6-phosphate and pryruvate levels, a reduction of metabolites associated with the TCA cycle was measured in the bevacizumab-treated tumors. These included pyruvate, *cis*-aconitate, α-ketoglutarate, succinate, fumarate and malate. Moreover, reduced levels of l-glutamine were observed following bevacizumab treatment. **b** Bevacizumab treatment led to reduced levels of glutathione and metabolites associated with glutathione synthesis, including l-cysteine, l-glutamate and l-glycine

Based on the comprehensive datasets obtained by LC–MS analysis, we also determined the metabolite quantities associated with the TCA cycle. Figure 4 shows the total level (^12^C unlabeled as well as ^13^C labeled) of key metabolites. In addition to a reduction in total glucose, glucose-6-phosphate (G6P) and pyruvate in the bevacizumab-treated tumors, we observed a significant reduction in cis-aconitate, α-ketoglutarate, succinate, fumarate and malate (Fig. 4a). The results were similar for labeled and unlabeled metabolites indicating a strong correlation between these subpopulations (Supplementary Fig. 3). Changes in these metabolites were also seen in the contralateral brain tissue, again suggesting that bevacizumab may also induce metabolic changes in the normal brain, although this observation could also be explained by infiltrating tumor cells. In agreement with the flux analysis, these data indicate an uncoupling of glycolysis from the TCA cycle in favor of increased lactate production.

Interestingly, the data also revealed reduced levels of l-glutamine upon treatment, suggesting that glutamine may be used to refuel the TCA cycle as a result of treatment (Fig. 4a).

Finally, the LC–MS analyses indicated that bevacizumab treatment led to a reduction in metabolites associated with the γ-glutamyl cycle, such as cysteine, glutamate and glycine, with a concomitant reduction in glutathione (Fig. 4b). Glutathione is known for its antioxidant properties by scavenging free radicals and reactive oxygen species; hence its depletion may be associated with increased oxidative stress. Since glutathione is recycled from oxidized glutathione via NADPH consumption, we asked the question whether NADPH levels were affected by the treatment. Although we detected much higher levels of NADPH in tumors compared to normal brain, we did not see any difference in the NADP^+^/NADPH ratio between treated and untreated tumors (Supplementary Fig. 4).

In summary, the results from this in depth metabolomic analysis strongly suggest that bevacizumab treatment causes a shift from aerobic respiration to anaerobic metabolism. Moreover, reduced levels of glutathione indicate that the treatment may induce oxidative stress in the tumors.

### Lactate dehydrogenase (LDH) levels are increased in bevacizumab-treated tumors

**Fig. 5:**
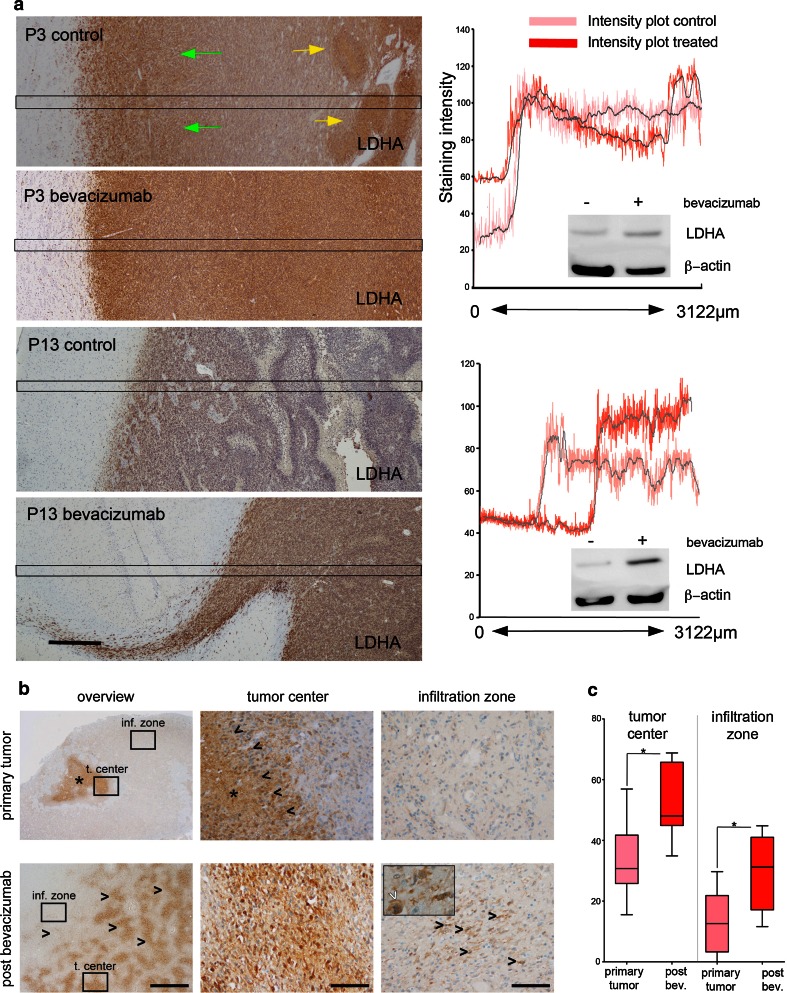
Bevacizumab treatment leads to an increased lactate dehydrogenase (LDH) expression. **a** Immunohistochemical analysis of P3 and P13 tumors show an increased expression of LDH in the bevacizumab-treated tumors (*bar* 500 µm). For the P3 control tumors, LDH was highly expressed in the periphery (*left panel*; *green arrows*) as well as in hypoxic pseudopalisading necrotic areas (*left panel*; *yellow arrows*). *Right panel*, quantification of LDH expression in control and treated tumors (quantification was made over defined area of 3,122 µm, outlined in the immunostained sections). Western blots from both tumors confirmed the immunohistochemical observations (*left panel*). **b** Immunohistochemical analyses of human primary GBMs predominantly exhibit strong LDHA expression in tumor cells surrounding necroses (*arrowheads upper middle*, *asterisks* indicating necroses), while infiltration zones were virtually devoid of LDHA-positive cells. After bevacizumab treatment the LDHA expression pattern changed from a virtually exclusive perinecrotic pattern to a patchy leopard-skin-like expression (*arrowheads lower left*) also in non-necrotic tumor centers (*lower middle*) as well as in infiltration zones (*lower right*). Infiltrating pleomorphic tumor cells (*black arrowheads lower right*) strongly express LDHA (*white arrowhead* blowup on lower right indicating mitotic figure). (*t. center* tumor center, *inf. zone* infiltration zone; *scale bars*: lower left 1 mm, lower middle and right 100 µm). **c** Quantification of matched-pairs analyses revealed higher frequencies of LDH-positive cells in tumor centers (*p* = 0.0137) as well as in infiltration zones (*p* = 0.0159) after bevacizumab treatment. The number of LDHA-positive cells where quantified as fraction of the whole cell population

Since an increased accumulation of lactate was observed in the metabolic flux study, we studied the expression of the enzyme lactate dehydrogenase (LDH) in the P3 and P13 xenografts by immunohistochemistry and by Western blots. LDH5 is the hypoxia-inducible isoform of the tetrameric enzyme LDH, composed of four identical subunits transcribed from the LDH-A gene [46]. For the P3 control tumors, LDHA was highly expressed toward the periphery (Fig. 5a, green arrows) as well as in hypoxic pseudopalisading necrotic areas in the tumor core (Fig. 5a, yellow arrows). The staining was increased and appeared more homogeneous after treatment as determined by intensity plots (Fig. 5a, right panels). Also in the P13 tumors, a strong up-regulation of LDHA was seen after bevacizumab treatment. Western blots from both tumors confirmed the immunohistochemical observations (Fig. 5a, right panels).

To validate these observations in clinical specimens, we obtained tumor samples from eight GBM patients before and after bevacizumab treatment (see Supplementary Table 1 for patient characteristics).

Immunohistochemistry analysis of tumor tissue obtained before bevacizumab treatment showed LDHA to be mainly expressed by tumor cells located in perinecrotic areas (Fig. 5b) and to a lower extent in tumor cells outside these areas. In post-treatment samples, however, the LDHA expression changed into a patchy “leopard-skin”-like staining pattern displaying the highest frequency of LDHA-positive cells in the tumor center, but also a strong expression by diffusely infiltrating tumor cells (Fig. 5b). Matched-paired analyses of the patients revealed a significant increase of LDHA-positive cells in tumor centers as well as in infiltration zones after bevacizumab treatment (Fig. 5c). Since some tumors were taken from biopsies and others from autopsy material, we performed ANOVA analysis of LDHA expression after bevacizumab treatment with regard to biopsy vs. autopsy. This revealed no statistical difference between both conditions, in the infiltration zone (*p* = 0.9838) or in the tumor center (*p* = 0.5826) (Supplementary Fig. 5). In the infiltration zone, mean LDHA % in the biopsy group was 29.53 (CI 10.617–48.45, median 35.2) and mean LDHA % in the autopsy group was 29.74 (CI 15.087–44.39, median 27.2). In the tumor center, mean LDHA % in the biopsy group was 49.10 (CI 31.046–67.154, median 45.8) and mean LDHA % in the autopsy group was 29.74 (CI 15.087–44.39, median 27.2) (Supplementary Fig. 5a). No correlation was found between LDHA expression and last bevacizumab time to histology (*r* = 0.098; *p* = 0.8154), in the tumor center or in the infiltration zone (*r* = −0.179; *p* = 0.6708) (Supplementary Fig. 5b). Immunofluorescence double staining with antibodies against CD68 ruled out that microglial cells were the source for LDH expression in the infiltration zone (Supplementary Fig. 6).

In summary, the increased expression of LDH together with the increased levels of lactate suggests that bevacizumab treatment leads to an acidification of the microenvironment caused by glycolysis. This acidification may not only occur in the tumor core, but also in the infiltrative compartment of GBMs.

### Flow cytometric phenotyping indicates that the change in phenotype represents an adaptive response and is not caused by clonal evolution and cancer stem-like cell selection

**Fig. 6:**
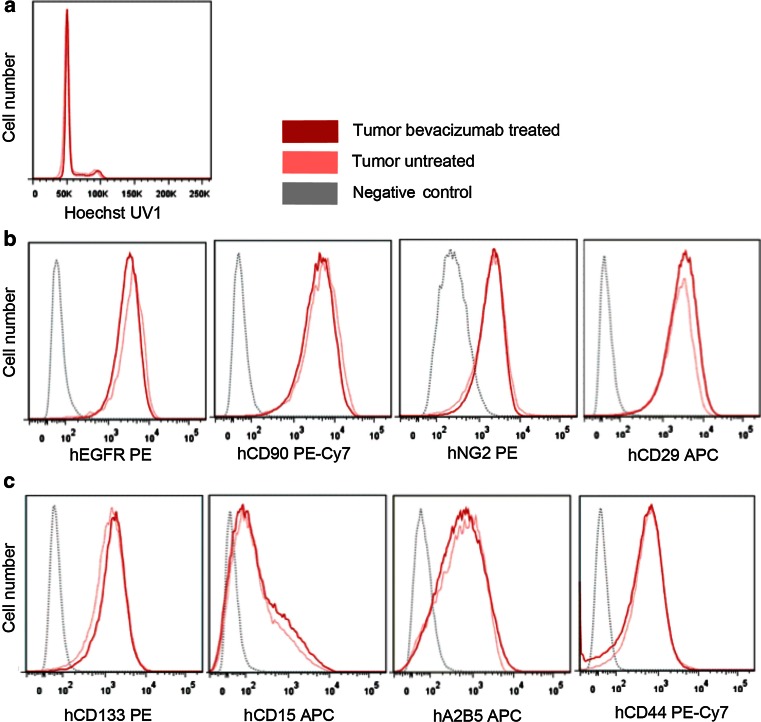
Flow cytometric analysis indicates that the change in phenotype represents an adaptive response and not clonal evolution and stem-like cell selection. **a** Hoechst-based ploidy measurements revealed that pseudodiploid P3 xenografts retained their initial ploidy after 3 weeks of bevacizumab treatment. **b** Flow cytometric phenotyping of tumor cells revealed no change in expression levels of classical glioma tumor cell membrane proteins such as EGFR, CD90, NG2 and CD29. **c** Also expression of the putative glioma stem cell markers CD133, CD15, CD44, A2B5 remained the same after treatment

An inevitable relapse of disease upon treatment may appear due to genetic or phenotypic cellular heterogeneity present within the tumor bulk [18, 41]. We have recently shown that GBMs display strong heterogeneity at the DNA ploidy level and at the level of stem cell marker expression [53]. We also found that aneuploidization is a late event in GBM development and that aneuploid cells show differential growth characteristics compared to pseudodiploid clones [53]. To assess whether bevacizumab treatment leads to a selection of specific cell types within the tumors, we analyzed ploidy and copy number aberrations of tumor cells following treatment. To this aim, P3 spheroids were implanted intracranially in eGFP^+^ NOD/Scid mice [40], and tumor cells were recognized as GFP negative cells (Supplementary Fig. 7). Hoechst-based ploidy measurements revealed that pseudodiploid tumor cells retained their initial ploidy upon bevacizumab treatment (Fig. 6a). Moreover, aCGH-based genomic analysis showed no new genetic aberrations appearing following treatment (data not shown).

Phenotypic heterogeneity has been associated with the presence of putative cancer stem-like cells that have been proposed to be responsible for drug resistance and tumor relapse [55]. To assess whether anti-angiogenic treatment selects for a particular subpopulation of tumor cells, we analyzed expression profiles of cell membrane markers known to be expressed in GBM cell subpopulations. Flow cytometric analysis of the tumor cells revealed no change in expression of classical tumor membrane proteins such as EGFR and CD29 (Fig. 6b). Similarly, expression profiles of putative stem cell markers including CD133, CD15, CD44, A2B5 were unchanged upon treatment (Fig. 6c), suggesting no selection for a particular tumor cell subtype and no adaptation toward a more stem-like phenotype.

In summary, we show that the genomic and stem cell-associated profiles of the tumor cells remain stable after bevacizumab treatment, suggesting that the observed metabolic changes occur as a result of an adaptive response of tumor cells, rather than from a selection of a particular subpopulation with increased resistance to treatment.

## Discussion

Angiogenesis is a prominent feature in GBMs where an activation of the VEGF-A signal transduction pathway, mediated by autocrine and paracrine mechanisms, represents a prominent component of angiogenesis induction [45]. Based on promising phase II studies that suggested a reduction in tumor size and prolongation of progression-free survival with radiological response rates ranging between 21 and 61 % for recurrent GBMs [28, 56], two large phase III clinical trials were initiated (AVAglio and RTOG 0825). Despite minor differences, the two trials showed that bevacizumab treatment had no effect on overall patient survival, but with a somewhat longer progression-free survival [6, 15]. Several mechanisms have been proposed on how tumors may resist anti-angiogenic therapy (reviewed in [32, 33, 51], yet the mechanisms behind this refractoriness are unclear. Preclinical models of other cancers suggest that antiangiogenic therapy may cause a temporary vascular normalization leading to increased blood perfusion and improved oxygen delivery [25]. Initial studies on GBMs with cediranib, a pan-VEGF receptor tyrosine kinase inhibitor, showed structural normalization of blood vessels and reduced vessel leakage [2]. However, it was later found that only a small percentage of patients (7 out of 30) displayed a functional normalization of the vessels with increased blood perfusion [52]. We have previously shown in a preclinical model that characterizes human GBMs, that bevacizumab treatment causes an induction of hypoxia leading to reduced tumor perfusion [27]. The patient-derived xenograft models used in these studies have previously been shown to retain the histological features of human GBMs, including infiltrative growth, angiogenesis and necrosis, while maintaining the heterogeneity of tumor subpopulations and the genetic mutations of the primary tumor [16, 49, 58]. We therefore chose to use these models instead of syngeneic models or xenogeneic models established from cell lines that frequently show genetic drift and clonal selection resulting in the loss of key histological and genomic features that characterize human GBMs [30]. In the present work, our previous observations on tumor hypoxia are further substantiated by in vivo ^18^F-FMISO PET imaging showing a gradual increase in hypoxia following treatment (Fig. 2c). This phenomenon has also been observed in the clinic [11, 20] where it has been shown to have a predictive value on patient survival after treatment onset [50]. Even though strong induction of hypoxia has been observed by us and other groups, it cannot be ruled out that an increased perfusion may occur locally or in a subset of patients following treatment. Although it is presently not clear if this phenomenon also occurs in bevacizumab-treated patients, it will be interesting to determine the biomarker potential of oxygenation in GBM patients as has been suggested for cediranib [1].

To determine how GBMs adapt to bevacizumab treatment, we performed a detailed ^13^C_6_-glucose metabolic flux analysis comparing untreated and treated tumors. Using glucose labeled with ^13^C on all the carbons it has been shown in glioma patients that glucose is cleaved glycolytically for lactate production, but also that an important fraction of glucose carbons enter via pyruvate into the TCA cycle under physiological conditions [36]. Thus, the oxidative mitochondrial metabolization of glucose is important in GBM for energy metabolism and to supply TCA-derived precursors for the synthesis of non-essential amino acids. We show that anti-angiogenic treatment leads to an increased influx of ^13^C_6_-glucose into the tumors with a subsequent increase in LDH and ^13^C-labeled lactate levels, while key metabolites associated with the TCA cycle are reduced following treatment. These data provide conclusive evidence that bevacizumab leads to hypoxic tumors with an increase in glycolytic activity and decreased oxidative respiration. The LC–MS analysis also revealed reduced levels of l-glutamine. These observations are in agreement with previous reports indicating that under hypoxia glutamine is used via reductive glutamine carboxylation to fuel macromolecule synthesis, in particular lipogenesis [38].

Interestingly, we also observed reduced levels of l-cysteine and l-cystathione as well as glutathione, suggesting increased oxidative stress in the tumors following bevacizumab treatment. Since the NADP^+^/NADPH ratio in the tumor was unchanged following treatment, the reduction in glutathione may be related to reduced de novo synthesis, as suggested by the changes in the carboxyl cycle metabolites. Maintenance of NADPH status is supported by our finding that key PPP enzymes are up-regulated following treatment, suggesting that this pathway is engaged to maintain NADPH and biomass production and ensure continued tumor growth even at low oxygen levels.

A previous study by Kathagen et al. [26] highlighted the metabolic flexibility of GBM cells, describing a switch between glycolysis and PPP activity following changes in environmental oxygen levels. In apparent contrast to our results, the group reported a down-regulation of PPP enzymes in response to severe hypoxia. However, it should be noted that the study investigated the response to acute changes of oxygenation, while our results are based on chronic hypoxic stress in vivo as also confirmed by ^18^F-FMISO imaging. Thus, it is plausible that an initial down-regulation of the PPP in response to acute or short-term hypoxia is followed by increased PPP activity upon adaptation of the tumor cell to hypoxic conditions.

A key question is why the GBMs in our study turn to glycolysis following bevacizumab treatment that will give a growth advantage. In this context, a common property of many cancers is up-regulation of glycolysis, resulting in increased glucose consumption, frequently seen in clinical tumor imaging. It has therefore been suggested that a persistent turnover of glucose to lactate even under aerobic conditions (Warburg effect) is an efficient means to increase biomass production and maintain high proliferation rates [14]. The up-regulation of glycolysis and the induction of microenvironmental acidosis after bevacizumab treatment may lead to a cellular adaptation, on the one hand maintaining continued tumor growth and on the other hand promote invasion. Such adapted cells have a powerful growth advantage, which may promote unconstrained proliferation and invasion. Support for this view comes from intravital microscopy studies showing increased tumor cell invasion in areas with low pH levels in experimental models [10]. Also in our xenografts models, we observed an up-regulation of LDH and increased lactate levels suggesting an acidification of the microenvironment. Interestingly, single invasive LDH-positive tumor cells were observed even in highly oxygenated normal brain areas following treatment, a phenomenon that was also observed in patient biopsies taken at various time points after treatment (Fig. 5). Thus, our findings were also validated in human biopsies. Similar results were recently reported in another study [19] in which lactate levels were assessed in vivo using multivoxel ^1^H MR spectroscopy. Elevated levels of lactate were reported in the tumor core. Yet, in the diffuse infiltrative areas, the levels of lactate were lower, possibly reflecting that the limited fraction of infiltrated cells in healthy tissue is insufficient to significantly influence the lactate level measured at the scale of MRS voxels (mmol). It should, however, be emphasized that under normoxia, the LDH-positive infiltrative cells, may convert lactate to pyruvate which may enter the TCA cycle. It is therefore still an open question if the infiltrative tumor cells are truly glycolytic.

To evaluate whether the changes observed after treatment could be explained by a selection of a new genetic clone and/or putative stem cell-like populations with increased resistance to treatment, we performed flow cytometric ploidy and multiparameter phenotypic marker studies on non-treated and treated tumors. We did not observe any changes in ploidy levels, genetic aberrations and stem-like associated cell membrane marker profiles following bevacizumab therapy, suggesting that the change in cellular behavior after treatment represents an adaptive metabolic response. Yet, further studies are warranted to delineate in detail to what extent clonal subpopulations within GBMs contribute to the changes in metabolic phenotypes observed.

Our findings of increased intratumoral hypoxia and glycolytic activity, leading to increased acidification of the microenvironment and invasiveness, suggest that therapies that target the metabolic adaptation mechanisms may provide a synergistic effect on bevacizumab therapy. Indeed, a number of preclinical studies have started to show reduced tumor progression when bevacizumab is combined with inhibitors targeting evasion mechanisms related to invasion and metabolism [5, 7, 22, 24, 29, 34, 37, 39, 42].

In conclusion, we show that bevacizumab treatment causes a metabolic shift in the tumors toward glycolysis where the cells depend less on oxidative respiration to produce ATP. This makes the recurrent tumor cell populations less dependent on angiogenesis. Moreover, this shift supports the notion that H(+) diffuses from the proximal tumor microenvironment into adjacent normal tissues where it causes tissue remodeling that permits local invasion. In this context a future challenge will be to develop therapeutic approaches that target adaptive metabolic responses in tumors as a result of therapy-induced perturbations.

## Electronic supplementary material

Below is the link to the electronic supplementary material.

Supplementary material 1 (DOCX 3766 kb)
