# Supplementary material for: Bevacizumab treatment induces metabolic adaptation toward anaerobic metabolism in glioblastomas
Source: Acta Neuropathol. 2014 Oct 17;129(1):115–31. doi: 10.1007/s00401-014-1352-5 (PMC4282692; doi:10.1007/s00401-014-1352-5)
Supplement: Supplementary file 1 — Supplementary material 1 (DOCX 3766 kb) [file 401_2014_1352_MOESM1_ESM.docx]

**Supplementary material**

**Supplementary figure 1**


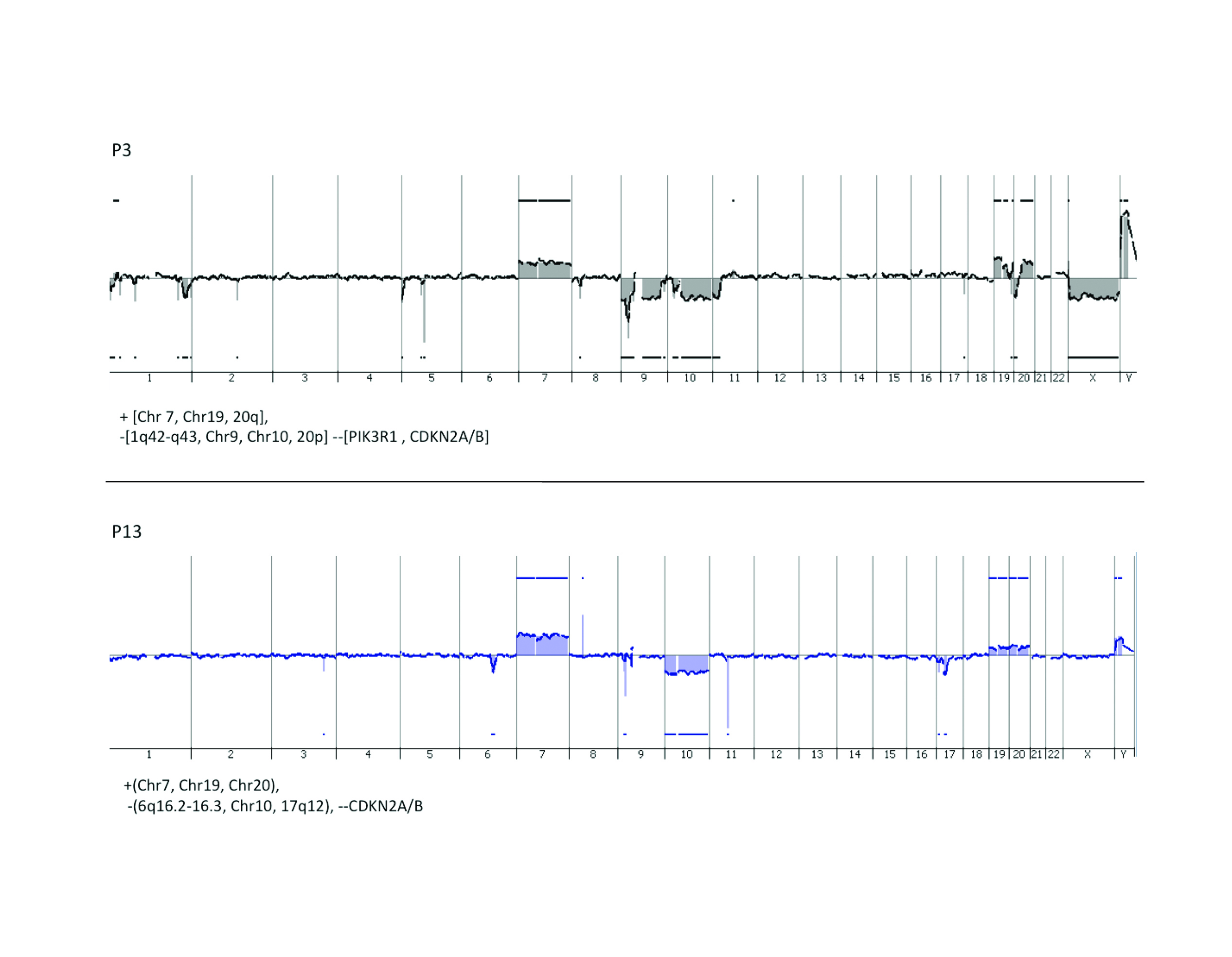


Array CGH profiles of the P3 and P13 human GBM xenografts, showing typical DNA copy number aberrations shown in human GBMs. P3: amplification of Chr 7, Chr19, 20q, and deletions of 1q42-q43, Chr9, Chr10, 20p, PIK3R1 , CDKN2A/B and for P13: amplification of Chr7, Chr19, Chr20 and deletions of 6q16.2-16.3, Chr10, 17q12, and CDKN2A/B. No changes in the aCGH profiles were observed following treatment (data not shown).

The procedure for the aCGH analysis was as follows: Genomic DNA was extracted from P3 and P13 xenografts using the DNAeasy Blood and Tissue Kit (Qiagen) following the manufacturer’s instructions. DNA was eluted in water, fragmented to an average size of 200-500 bp using DNAse1 (rDNAse1, Ambion, Life Technology Ltd.) and labeled using the BioPrime aCGH Genomic labeling Kit (Invitrogen) and Cy3 and Cy5 dyes purchased from GE Healthcare (Chalfont St. Giles, UK) following standard protocols for Agilent aCGH. Commercially available female DNA pooled from multiple anonymous donors (Promega, Madison, WI) was used as a reference for each of the aCGH experiments. Labeled DNA was competitively hybridized to SurePrint G3 Human 2x400k CGH microarrays (Agilent Technologies, Santa Clara, CA) following standard Agilent protocols. The slides were scanned at 3µm resolution using the Agilent High-Resolution Microarray scanner and the image data were extracted using Feature Extraction (Agilent Technologies). FE extraction files were imported into Genomic Workbench 7.0 (Agilent Technologies) for visualization and analysis. Aberrations were called using the Aberration Detection Method 2 (ADM2) algorithm with a threshold setting of 25, centralization on with threshold of 25 and an aberration filter min Probes=3 and minAvgAbsLogRatio=0.25. The ADM-2 algorithm identifies all aberrant intervals in a given sample with consistently high or low log ratios based on the statistical score that represents the deviation of the average of the log_2_ ratios from the expected value of zero.

**Supplementary figure 2**


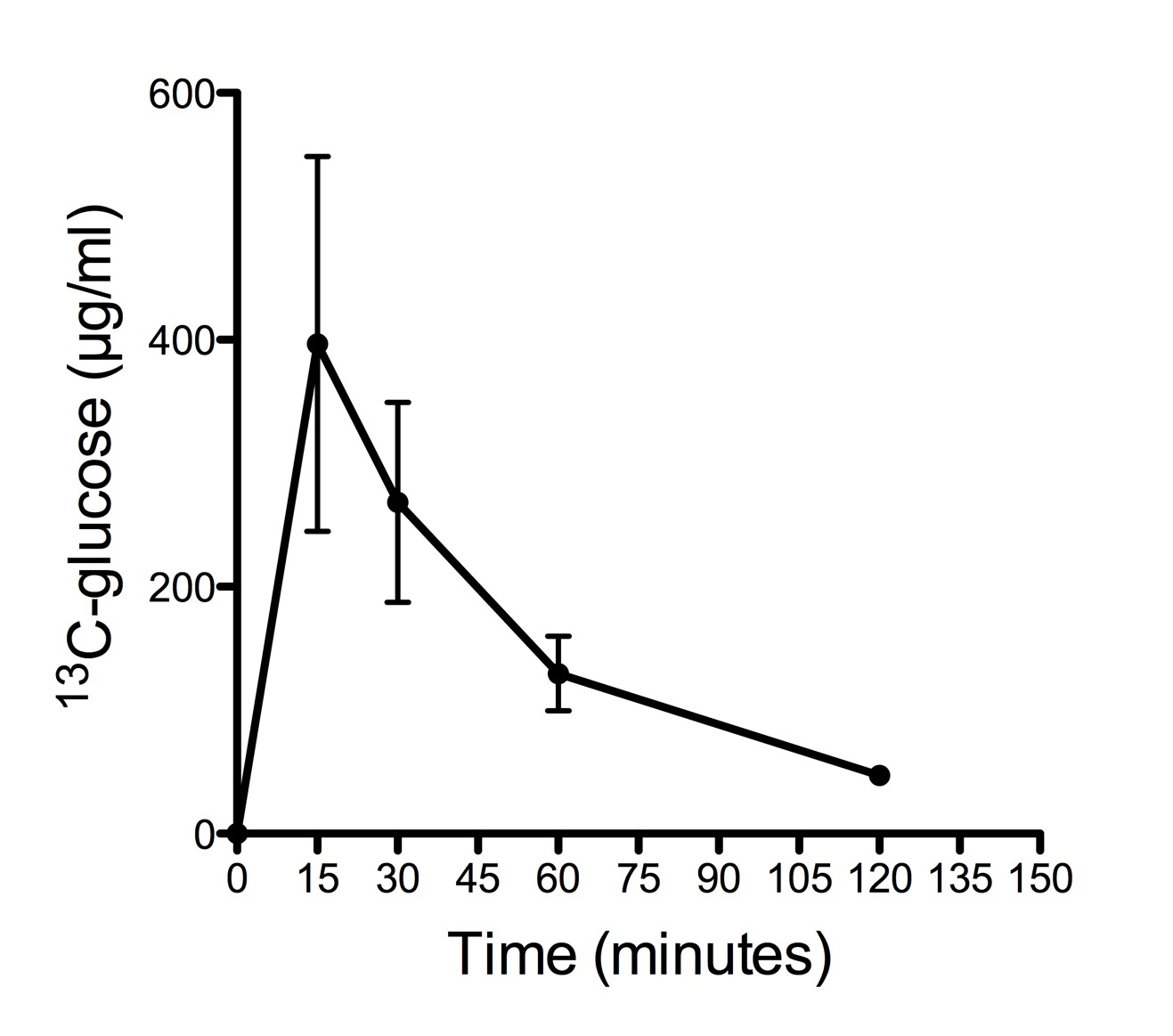


Three normal rats were injected with a dose of 0.5ml/100g body weight using a 200mg/ml ^13^C6-labeled glucose solution. Blood samples were collected after 15, 30, 60 and 120min. The figure shows the serum concentrations of ^13^C6-labeled glucose as detected by LC-MS analysis (Methods described in main text).

**Supplementary figure 3**


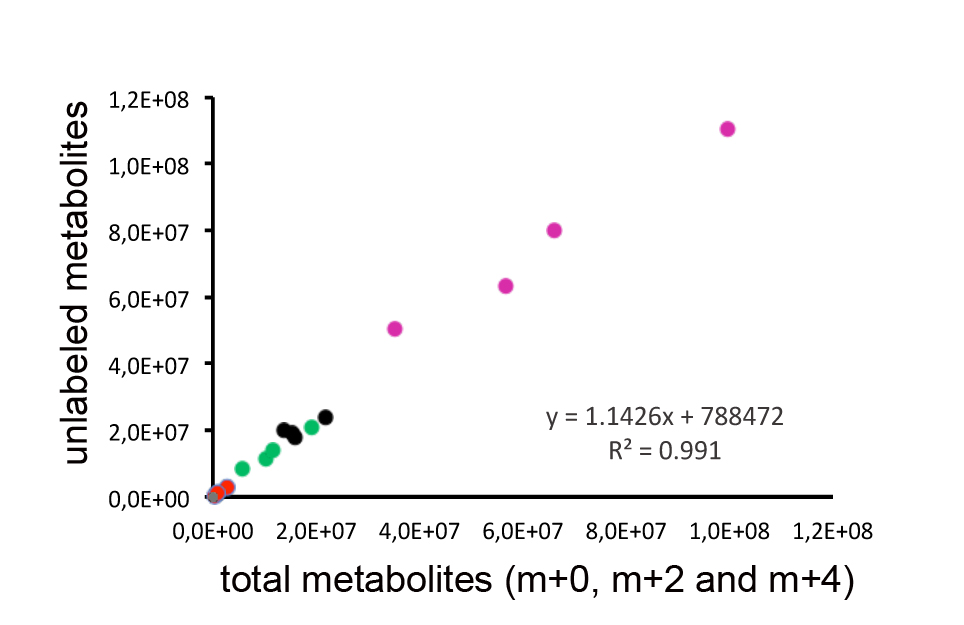


A strong correlation (R^2^= 991) was observed between the unalabeled and total metabolites (m+0, m+2 and m+4). Pink: malate, Black: succinate, Green fumarate, Red: α−ketoglutarate, Grey: cis-asconitate.

**Supplementary figure 4**

a

b

Tissue extracts from contralateral brain (B; n=3) and xenografted tumors (T; n=4) from control (0) and bevacizumab (1) treated rats sacrificed 15 minutes after glucose administration were analyzed, as well as a control brain from healthy rats without tumor implantation (B_ctrl). (a) NADP^+^/NADPH ratios in indicated samples, as compared to healthy rat brain. A higher ratio was observed in contralateral brain samples compared to tumor, but no significant difference between treated and untreated samples was detected. (b) NADPH quantities were extrapolated from the calibration curve and show that in tumor extracts, NADPH is more abundant than in contralateral and control brain, which explains the higher ratio seen in (a).

**Supplementary figure 5**


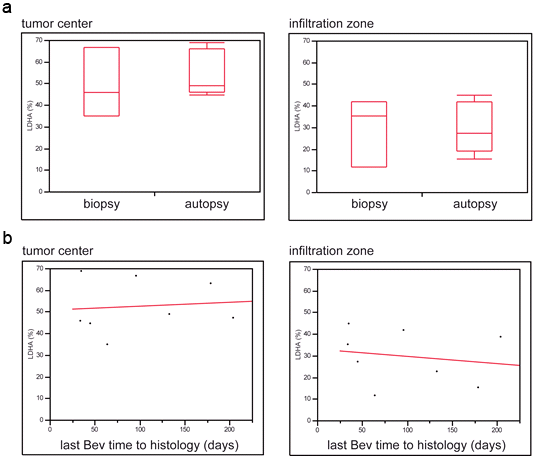


Influence of tissue collection approach and delay from last bev treatment on LDHA level. (A) ANOVA analysis of post-bev LDHA expression with regard to tissue collection approach (biopsy vs. autopsy) did not reveal statistically significant differences, neither in tumor center (p=0.9838) nor in the infiltration zone (p=0.5826). (B) Correlation analysis between LDHA expression and last bev time to histology shows no correlation between the variables, neither in tumor center (r=0.098; p=0.8154) nor in the infiltration zone (r=-0.179; p=0.6708).

**Supplementary figure 6**

**
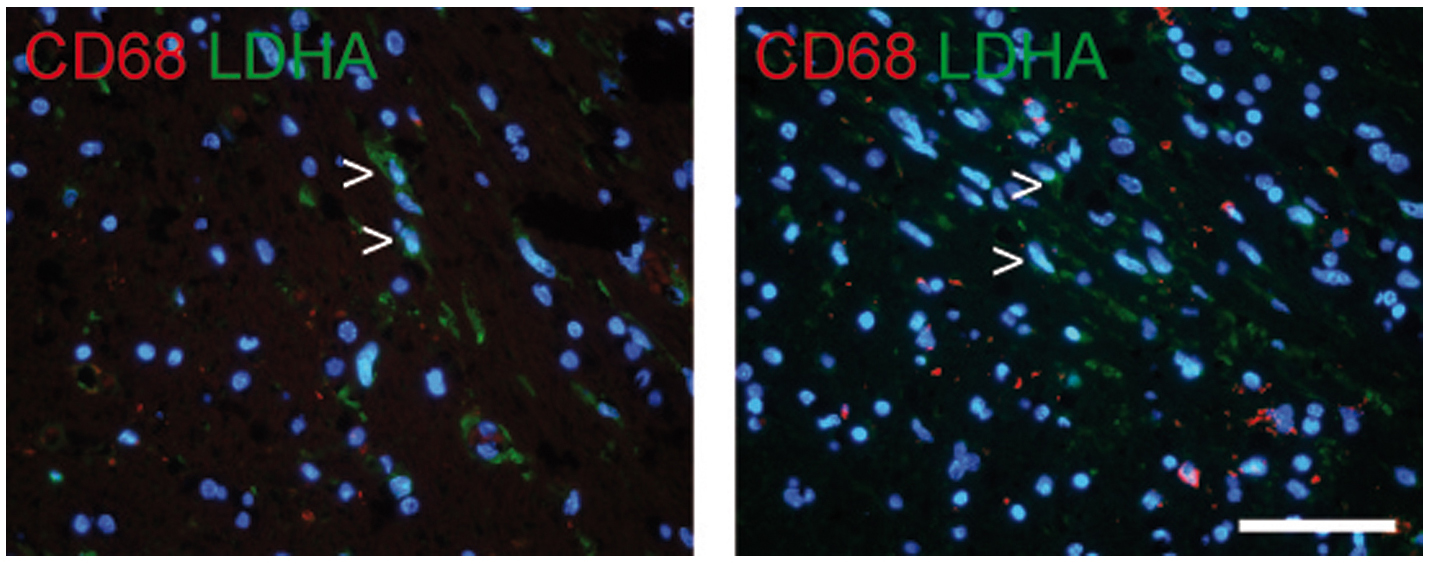
**

Immunofluorescence double staining ruling out that CD68 positive microglial cells are a source for LDHA expression. Arrowheads point at LDHA-positive spindle shaped pleomorphic cells in the infiltration zone of a post-bevacizumab patient, while CD68-positive microglia are LDHA negative (scale bar 50µm). Methods described in main text.

**Supplementary figure 7**


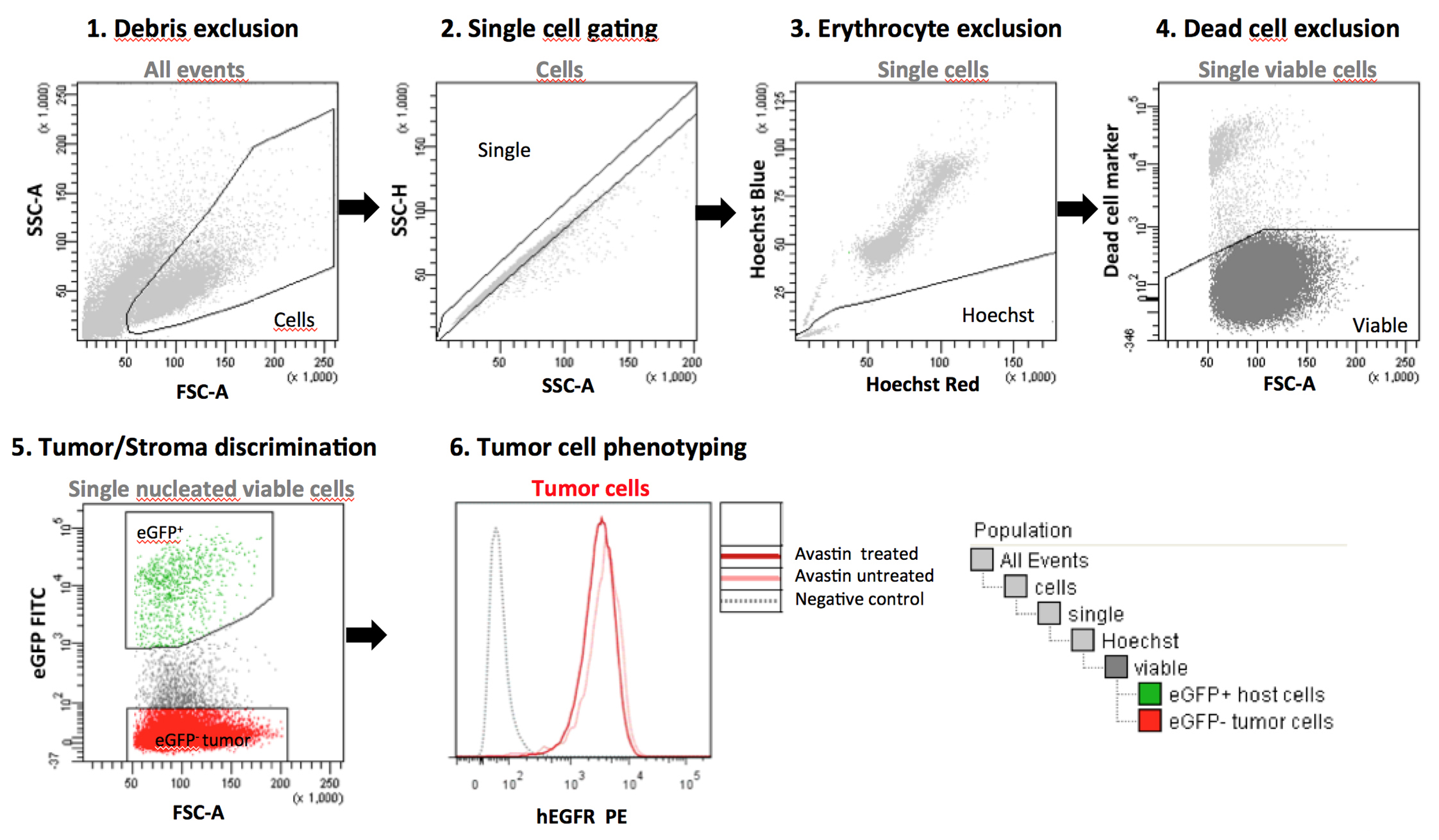


Gating strategy for sorting and multicolor phenotypic analysis. The step by step gating strategy for FACS analysis is shown for the intracranial P3 xenograft in eGFP^+^ NOD/SCID mice. (**1)** Cells were distinguished from debris on the flow cytometric profile based on the Forward Scatter (FSC) and Side Scatter (SSC). (**2)** Cell doublets and aggregates were gated out based on their properties displayed on the SSC area (SSC-A) versus height (SSC-H) dot plot. (**3)** Erythrocytes were excluded by applying a ‘Hoechst’ gate on the ‘Hoechst Red’/’Hoechst Blue’ dot plot in the linear scale. (**4)** Dead cells were recognized by their strong positivity for the dead cell discrimination marker. (**5)** In xenografts, human tumor cells were recognized as the eGFP negative population (red) compared to the eGFP positive mouse stromal host cells (green). **(6)** Multicolor phenotyping in tumor compartment of the xenografts was performed with human-specific antibodies. An example is shown for human specific EGFR staining in tumor cells in bevacizumab treated and control animals versus unstained negative control.

**Supplementary Table I, Human glioblastomas, patient characteristics**

| *Patient ID* | *age at primary tumor (years)* | *sex* | *MGMT*  *status* | *bev treatment (days)* | *last bev time to histology (days)* | *Primary tumor* | *Post bev* |
| --- | --- | --- | --- | --- | --- | --- | --- |
| ID1 | 51 | m | U | 91 | 179 | B/R | A |
| ID2 | 30 | m | U | 407 | 204 | B/R | A |
| ID3 | 52 | m | U | 84 | 35 | B/R | A |
| ID5 | 56 | m | n.k. | 288 | 45 | B/R | A |
| ID6 | 47 | m | U | 214 | 133 | B/R | A |
| ID10 | 63 | m | U | 336 | 64 | B/R | B/R |
| ID12 | 70 | f | U | 258 | 34 | B/R | B/R |
| ID14 | 46 | f | U | 170 | 96 | B/R | B/R |

All primary tumors were diagnosed as glioblastomas according to WHO grade IV. MGMT promoter methylation status is depicted as U (unmethylated) or n.k. (not known). The time from first until last bevacizumab administration is displayed as “bev treatment (days)” whereas the next column shows the time after last bevacizumab administration to final post-bevacizumab histology. In the last 2 columns B/R indicates that tissue was obtained from “biopsy/resection”, while A indicates that it was obtained from “autopsy”

**Supplementary Table II , List of antibodies used in the flow cytometry study.**

| **Epitope** | **Conjugate** | **Species reactivity** | **Clone** | **Supplier** | **Concentration used/test*** |
| --- | --- | --- | --- | --- | --- |
| A2B5 | APC/PE | human, mouse | 105-HB29 | Miltenyi | 10µl/test |
| CD15/SSEA-1 | Alexa Fluor 647 | human, mouse | MC-480 | Biolegend | 5µl/test |
| CD29 | APC | human | MEM-101A | Immunotools | 10 µl/test |
| CD44 | PE-Cy7 | human, mouse | IM7 | eBioscience | 1.2µl/test |
| CD90 | APC | human | 5E 10 | BD Bioscience | 5µl/test |
| CD133 | PE | human | AC133 | Miltenyi | 10µl/test |
| EGFR | PE | human | EGFR.1 | BD Bioscience | 20µl/test |
| NG2 | PE | human, mouse | LHM-2 | R&D | 10µl/test |

Flow cytometry test 10^6^ cells/100µl
